# Taxonomy of the genus *Poterioochromonas* (Chrysophyceae) based on morphological and molecular evidence

**DOI:** 10.1111/jpy.70028

**Published:** 2025-05-26

**Authors:** Minseok Jeong, Jong Im Kim, Woongghi Shin

**Affiliations:** ^1^ Department of Biology Chungnam National University Daejeon Korea

**Keywords:** Chrysophyceae, heterotrophic, mixotrophic, *Poterioochromonas*, stomatocyst, taxonomy

## Abstract

The genus *Poterioochromonas* is characterized by spherical cells having two unequal flagella, a greenish, yellow‐brown plastid, and a hemispherical cup‐shaped lorica with a long, narrow stalk and a foot anchoring to the substrate. In this genus, the only three mixotrophic species having plastids have been reported up to date: *P. malhamensis*, *P. nutans*, and *P. stipitata*. However, we observed heterotrophic *Poterioochromonas* species that had lost their plastids, and we successfully cultured them. To understand the taxonomy of *Poterioochromonas* species, we performed a molecular phylogenetic analysis and observed their morphological features using light and scanning electron microscopes. For the phylogenetic analysis, we used a combined dataset from five gene sequences: the nuclear small subunit (SSU) rRNA gene, the large subunit (LSU) rRNA gene, the internal transcribed spacer region (ITS rRNA region 1–5.8S‐ITS2), the plastid LSU rRNA gene, and the *rbc*L gene. The molecular phylogeny of the genus *Poterioochromonas* was divided into two major clades: mixotrophic and heterotrophic lineages. The mixotrophic clade comprised three species including two new species—*P. andersenii* sp. nov. and *P. longicaulis* sp. nov.—characterized by a colonial lifestyle and the long stalk of the lorica. The heterotrophic clade included the four new species—*P. amplexa* sp. nov., *P. communis* sp. nov., *P. similis* sp. nov.*,* and *P. sinechrysos* sp. nov.—that had lost their plastids. The species *P. amplexa* produced a very distinctive stomatocyst, which features a true complex collar. Here, we report six new species of *Poterioochromonas* that exhibit mixotrophy and heterotrophy, showing that the phylogenetic tree is distinctly divided according to nutritional modes.

**Abbreviations**
bp: base pairs
CW‐Eb: Calcofluor White‐Evans blue
DAPI: 6‐diamidino‐2‐phenylindole
DIC: differential interference contrast
LB: Luria‐Bertani
MC^3^
: Metropolis‐coupled Markov chain Monte Carlo
ML: maximum likelihood
MLBS: maximum‐likelihood bootstrap
NCMA: National Center for Marine Algae and Microbiota
nr: nuclear
PCR: polymerase chain reaction
PP: posterior probability
pt.: plastid

## INTRODUCTION

The genus *Poterioochromonas*, established by Scherffel (1901), is characterized by an *Ochromonas*‐like cell with biflagella, a plastid, an anterior feeding basket, and a cup‐shaped lorica attached to the substrate by a more or less long, fine stalk. It is known to feed on various organisms, including bacteria, cyanobacteria, and microalgae, and has a cannibalistic nutrition mode (Ma et al., 2018; Peck, 2010; Sanders et al., 1990; Scherffel, 1901; Weisse & Moser, 2020; Zhang et al., 1996; Zhang & Watanabe, 2001). Scherffel (1901) initially included only one species, *P. stipitata*. Later, Jane (1944) added a new species, *P. nutans*, based on morphological characteristics such as a broader and shallower lorica, a single plastid, and the absence of a food vacuole. A few years later, Pringsheim (1952) described a new species, *Ochromonas malhamensis*, and that species was subsequently transferred to *Poterioochromonas* based on the fine structure of the cup‐shaped lorica and the presence of the stalk (Péterfi, 1969). Currently, the three species have solitary lifestyles: *P. malhamensis*, *P. nutans*, and *P. stipitata*.

The key morphological characteristics used to distinguish *Poterioochromonas* from other loricate Chrysophycean species are its cup‐shaped lorica, its long and fine stalk, and its mixotrophic mode of nutrition, with some species forming a feeding basket at the anterior part of the cell to capture prey (Jane, 1944; Ma et al., 2018; Péterfi, 1969; Scherffel, 1901). The lorica is divided into two parts: the upper part that encloses the cell and the lower part that connects to the stalk. When observed under a light microscope, there is a fine line between these two parts (Jane, 1944; Scherffel, 1901). The walls of the lorica and the stalk are composed of fibrillar bundles that contain chitin (Herth, 1980; Herth et al., 1977; Péterfi, 1969). Based on morphological features, the genus *Poterioochromonas* was considered to have close relationships with the genera *Anthrochrysis*, *Anthropyxis*, *Stokesiella*, and *Dinobryon* in earlier taxonomic studies (Bourrelly, 1957; Pascher, 1942; Péterfi, 1969; Scherffel, 1901). Those genera all contain loricate Chrysophycean algae. However, those genera are well distinguished by the specific detailed morphological features of their lorica. For example, *Dinobryon* has a vase‐shaped lorica, yet both *Anthrochrysis* and *Anthropyxis* have cup‐shaped loricae with ring‐like constrictions below the middle of the lorica (Ehrenberg, 1834; Pascher, 1942). *Stokesiella* has a cylindrical or ovate lorica, is colorless, and possesses stalks of varying lengths (Lemmermann, 1910; Stokes, 1885a, 1885b). Recent molecular phylogenetic analysis using the nuclear SSU rRNA gene has shown that *Poterioochromonas* is a separate clade from both *Ochromonas* and *Dinobryon* and is classified within the Ochromonadales in Chrysophyceae (Andersen et al., 2017).

According to Scherffel's (1901) original description of *Poterioochromonas*, cell division results in daughter cells that either have chromatophores or lack them, and it has been suggested that cells without chromatophores do not adversely affect viability. For this reason, we believe that at that time, Scherffel recognized that the genus *Poterioochromonas* could include individuals without chromatophores. In this study, we have established some colorless *Poterioochromonas* taxa from multiple samplings from the sediments of freshwater lakes, ponds, and wetlands. To understand the taxonomy of the genus *Poterioochromonas*, we investigated the morphological features and performed phylogenetic analyses using the nuclear SSU rRNA gene, the nuclear LSU rRNA gene, the nuclear ITS rRNA region, the plastid LSU rRNA gene, and *rbc*L gene sequences. Based on morphology and molecular data, we propose six new *Poterioochromonas* species.

## MATERIALS AND METHODS

### Cultures

Non‐photosynthetic *Poterioochromonas* strains were isolated from the sediments of various ponds, lakes, and wetlands. We performed single‐cell isolation using a Pasteur capillary pipette to establish unialgal cultures from subcultures. To make subcultures by the dilution method, 3 μL of field freshwater samples were mixed into 200 μL of AF‐6 medium (Andersen et al., 2005) supplemented with 0.1%, 0.2%, or 0.5% Luria‐Bertani (LB) medium (Miller, 1972). The LB broth was for bacterial growth. Bacteria from field freshwater samples were fed to *Poterioochromonas* cells as prey. As we observed the growth of *Poterioochromonas* cells, a single target cell was isolated and transferred to a new single‐well plate by micropipette. Finally, all strains were grown in AF‐6 medium with a mixture of freshwater LB broth and uncharacterized bacteria from field freshwater samples. Four *Poterioochromonas* strains (CCMP1862, CCMP2060, CCMP2740, CCMP3181) were obtained from the National Center for Marine Algae and Microbiota (NCMA) culture collection at Bigelow Laboratory. The non‐photosynthetic strains were maintained at 17°C in a dark culture chamber, and photosynthetic strains were maintained at 17°C on a 14:10 h light:dark cycle with a light intensity of 30 μmol photons· m^−2^ · s^−1^ supplied by cool‐white fluorescent tubes (OSRAM Korea Co., Ansan, Korea). Details on the collection sites and the Genbank accession numbers for each strain are listed in Table S1.

### Light and fluorescence microscopy

**FIGURE 1:**
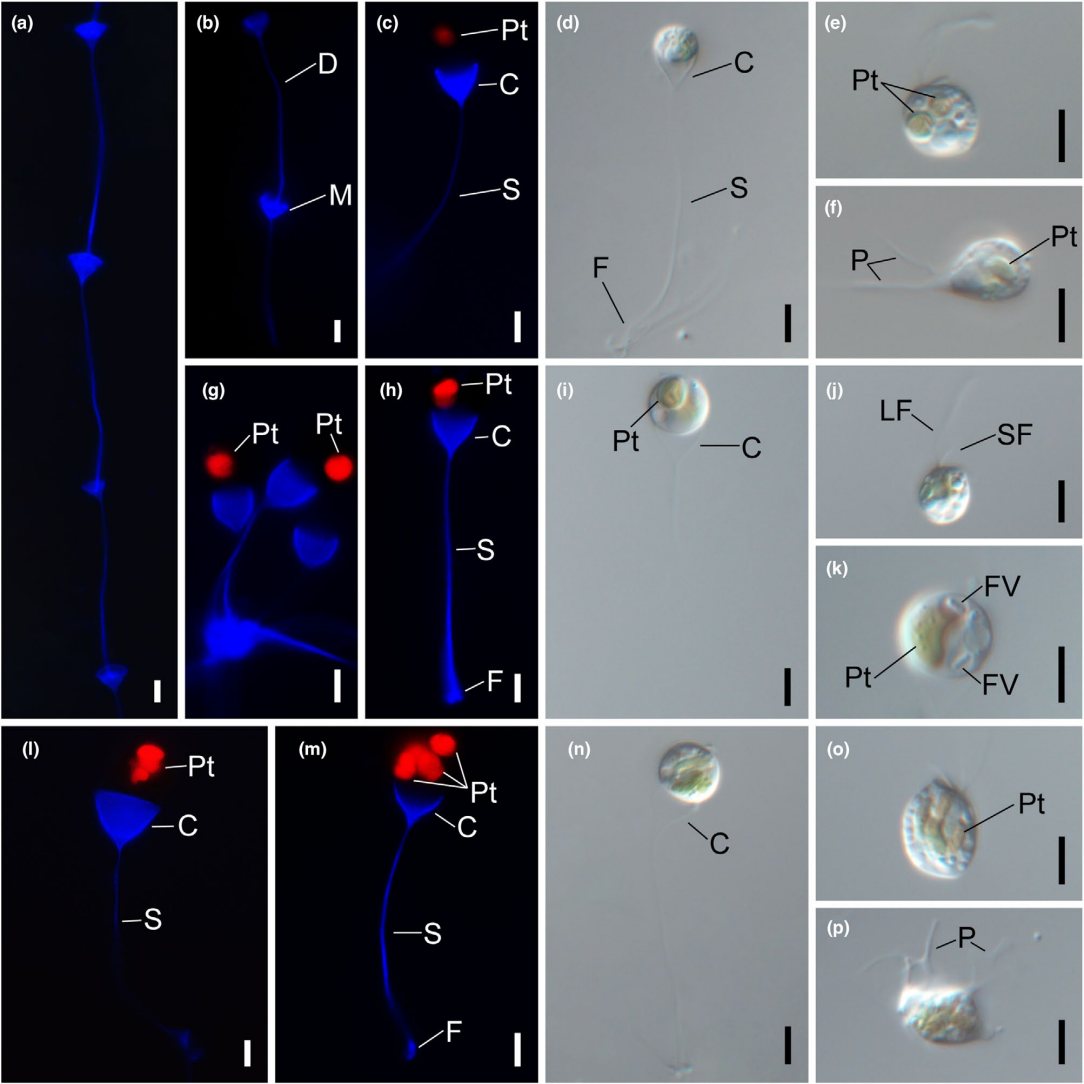
Light and fluorescence micrographs of photosynthetic *Poterioochromonas* species. Images showing a cell having a long (LF) and short flagella (SF) and plastids (Pt) contained in a lorica. The lorica is composed of a cup (C), stalk (S), and foot (F). The red autofluorescence originated from the plastids (Pt) and blue fluorescence originated from the lorica. (a, b) Colony morphologies of *Poterioochromonas andersenii* CCMP1862. The daughter lorica (D) attached their foot to inside the cup region of the mother lorica (M). (c, d) Lorica morphologies of *P. andersenii* CCMP1862. (e) Free‐swimming cell of *P. andersenii* CCMP1862. (f) Amoeboid stage of *P. andersenii* CCMP1862 with feeding‐basket (P). (g–i) Lorica morphologies of *P. longicaulis* CCMP2060. (j) Free‐swimming cell of *P. longicaulis* CCMP2060. (k) Food vacuole (FV) in cell of *P. longicaulis* CCMP2060. (l–n) Lorica morphologies of *P. malhamensis* CCMP2740. (o) Free‐swimming cell of *P. malhamensis* CCMP2740. (p) Amoeboid stage of *P. malhamensis* CCMP2740 with feeding‐basket (P). Scale bars = 5 μm.

**FIGURE 2:**
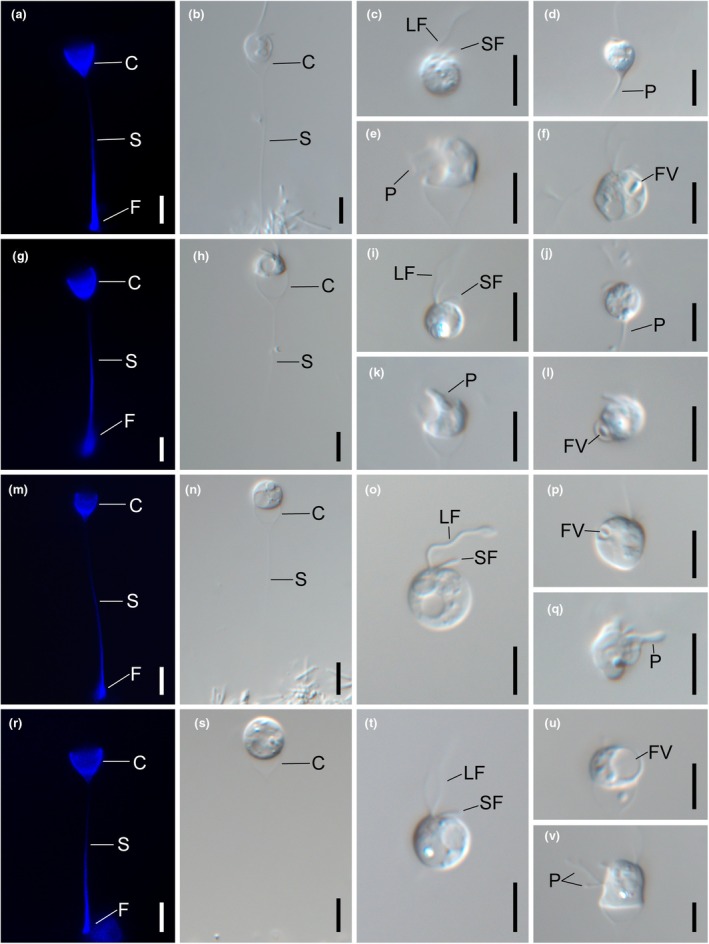
Light and fluorescence micrographs of non‐photosynthetic *Poterioochromonas* species. Images showing a cell having a long (LF) and short flagella (SF) contained in a lorica. The lorica is composed of a cup (C), stalk (S), and foot (F). The blue fluorescence originated from the lorica. (a, b) Lorica morphologies of *Poterioochromonas amplexa* Yeonso2je042421B9. (c) Free‐swimming cell of *P. amplexa* Yeonso2je042421B9. (d) Vegetative cell of *P. amplexa* Yeonso2je042421B9 attaching to substratum using feeding‐basket (P). (e) Amoeboid stage of *P. amplexa* Yeonso2je042421B9. (f) Food vacuole (FV) in cell of *P. amplexa* Yeonso2je042421B9. (g, h) Lorica morphologies of *P*. *communis* Ungok060918A1. (i) Free‐swimming cell of *P*. *communis* Ungok060918A1. (J) Vegetative cell of *P*. *communis* Ungok060918A1 attaching to substratum using feeding‐basket (P). (k) Amoeboid stage of *P*. *communis* Ungok060918A1. (l) Food vacuole (FV) in cell of *P*. *communis* Ungok060918A1. (m, n) Lorica morphologies of *P. similis* Widong120119A24. (o) Free‐swimming cell of *P. similis* Widong120119A24. (p) Food vacuole (FV) in cell of *P. similis* Widong120119A24. (q) Amoeboid stage of *P. similis* Widong120119A24 with feeding‐basket (P) (r, s) Lorica morphologies of *P*. *sinechrysos* Iljeong042421B4. (t) Free‐swimming cell of *P*. *sinechrysos* Iljeong042421B4. (u) Food vacuole (FV) in cell of *P*. *sinechrysos* Iljeong042421B4. (v) Amoeboid stage of *P*. *sinechrysos* Iljeong042421B4 with feeding‐basket (P). Scale bars = 5 μm.
The stomatocyst is spherical, ranges in size from 6.5–8.5 × 6.5–8.5 μm (*n* = 14), and has a smooth surface and complex collar (Figure 4). The cylindrical primary collar is surrounded by a secondary collar. The secondary collar is composed of a cylindrical collar base and two or three curved projections, ranging in height from 1.5 to 3.3 μm and from 2.3 to 3.1 μm (*n* = 14) in diameter. These projections converge at the apical end.
10 helix of V2 region from nr SSU rRNA: ACUCC
Loop region in 10 helix of V2 region from nr SSU rRNA: UGACUUCUGGAA
10′ helix of V2 region from nr SSU rRNA: GGAGU
E23‐5 helix of V4 region from nr SSU rRNA: GCGGC
E23‐5′ helix of V4 region from nr SSU rRNA: GCUGU
HOLOTYPE: NNIBR OR408, a permanent microscope slide prepared from strain Yeonso2je042421B9, deposited in the Nakdonggang National Institute of Biological Resources, Sangju, Korea (NNIBR)
ISOTYPE: NNIBR OR408, dried specimens placed on a filter paper (47 mm diam.).
Reference strain: Living material was deposited at the Korean Microalgae Culture Collection in Pukyong National University, Busan, South Korea as strain KMCC‐9001
TYPE LOCALITY: Eojeon‐ri, Geumsan‐myeon, Goheung‐gun, Jeollanam‐do, Korea (34.443386, 127.121251), 24 Apr. 2021
ETYMOLOGY: The Latin specific epithet “*amplexa*” refers to the secondary collar of the cyst surrounding the primary collar.

**FIGURE 3:**
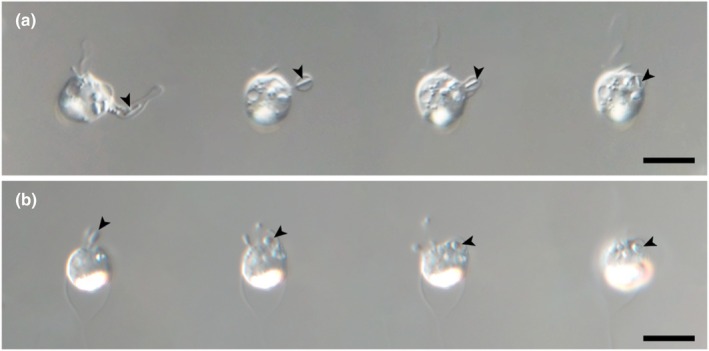
Images of two feeding behavior types of *Poterioochromonas amplexa*. (a) Captured images from video showing a feeding behavior using pronouncedly extended protrusion. (b) Serially captured images showing a feeding behavior using shallowly bulged protrusion. Arrowheads indicate bacteria. Scale bar = 5 μm.

Culture samples were observed using an Axio Imager A2 microscope (Carl Zeiss, Hallbergmoos, Germany) with differential interference contrast (DIC) optics. To observe loricae, *Poterioochromonas* species were stained with Calcofluor White‐Evans blue (CW‐Eb; Sigma‐Aldrich, St. Louis, MO, USA), in a 1:1 dilution of CW‐Eb in cultured *Poterioochromonas*. The 4′, 6‐diamidino‐2‐phenylindole (DAPI) filter and HPV 120 V (Carl Zeiss) were used for fluorescence microscopic observation of stained samples. Images were captured with an AxioCam 712 color (Carl Zeiss) photomicrographic system connected to the microscope (Figures 1, 2, 3). The terminologies used for morphological description followed those of Radford (1986).

### Scanning electron microscopy

**FIGURE 4:**
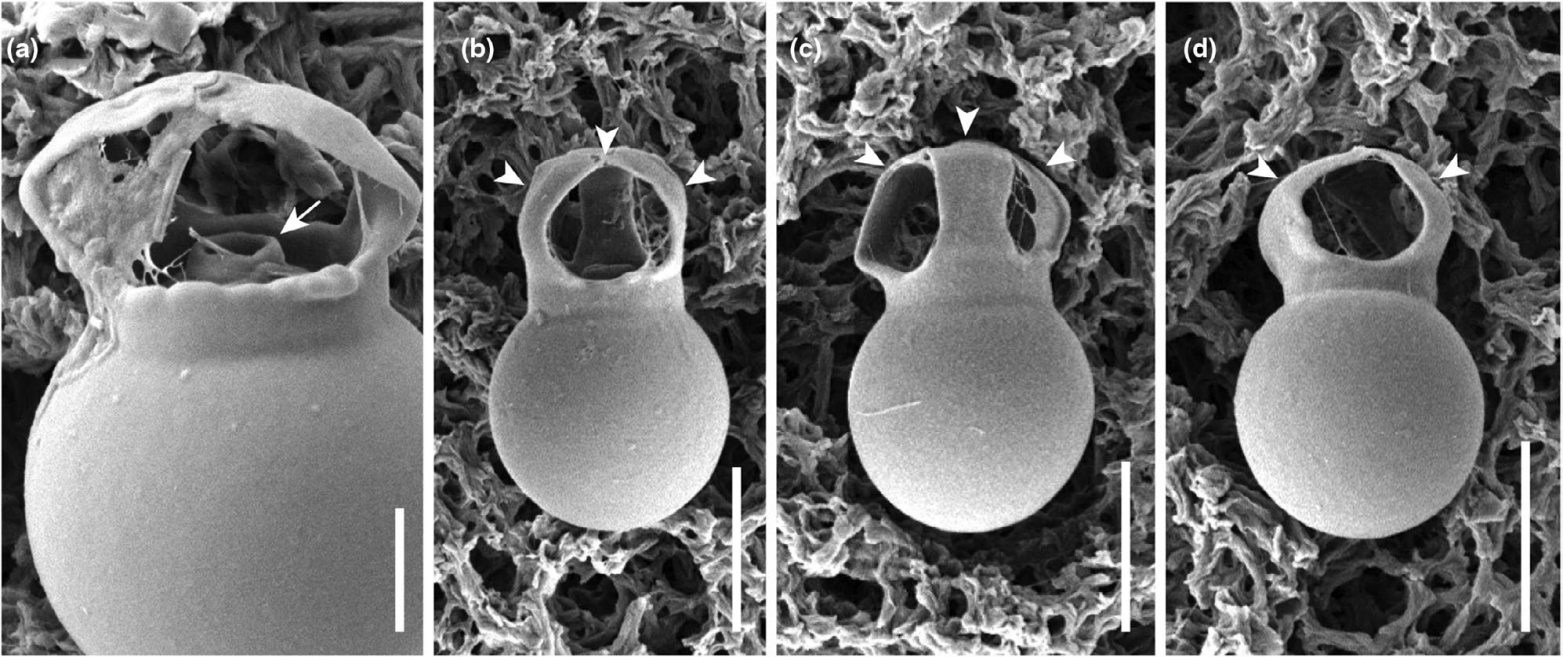
Scanning electron micrographs of *Poterioochromonas amplexa* stomatocyst. (a) The cylindrical primary collar (arrow) is surrounded by a secondary collar, (b–d) which is composed of a cylindrical base and two or three curved projections (arrowhead). Scale bars = 5 μm.

To observe stomatocyst morphology, encysted stomatocysts in cultures were collected by centrifugation at 9391 × *g* for 5 min (model 5424; Eppendorf, Hamburg, Germany) and boiled for 15 min in 10% HCl to remove the lorica. After washing three times with distilled water, stomatocysts were collected in a 1.5‐mL microcentrifuge tube and sonicated using an ultrasonic cleaner (Sae Han Ultrasonic, Seoul, Korea) to completely remove the lorica from the stomatocyst. The stomatocysts were mounted on a 0.45 μm nylon membrane. The filter was mounted onto aluminum stubs using double‐sided tape. The stubs were coated with platinum and viewed with a TESCAN CLARA field emission scanning electron microscope (TESCAN, Brno, Czech Republic) at 2 keV (Figure 4). The stomatocysts have been described according to International Statospore Working Group guidelines (Cronberg & Sandgren, 1986; Wilkinson et al., 2001).

### DNA extraction, amplification, sequencing, and sequence alignment

The culture cells were harvested by centrifugation at 9391 × *g* for 5 min (model 5424; Eppendorf). Genomic DNA extraction from culture cell pellets was performed using Exgene Cell SV following the protocol provided by the manufacturer (GeneAll Biotechnology, Seoul, Korea). The nuclear (nr) SSU rRNA gene, LSU rRNA gene, and ITS rRNA region and the plastid (pt) LSU rRNA and *rbc*L genes were amplified using specific primers as described in a previous study (Jeong et al., 2021, 2023). Polymerase chain reaction (PCR) was performed in a total volume of 25 μL consisting of the following: 1 μL of AccuPower® PCR premix (Bioneer Co., Daejeon, Korea), 1 μL of forward primer, 1 μL of reverse primer, 2–5 μL of template DNA, and 12–20 μL of distilled water. The genes were amplified using a T100™ Thermal Cycler (Bio‐Rad Laboratories, California, United States). The first denaturation was run at 94°C for 5 min, followed by 35 cycles of a second denaturation at 94°C for 30 s, annealing at 42–52°C for 30–60 s, extension at 60–72°C for 1–2 min, and a final extension at 60–72°C for 7 min, with a final hold at 12°C. All PCR products were purified using the Labopass™ PCR Purification Kit or Labopass™ Gel Purification Kit following the protocol provided by the manufacturer (Cosmogenetech, Seoul, Korea). The purified PCR products were sequenced using an ABI PRISM™ (3730xL; Perkin‐Elmer Applied Biosystems, Foster City, CA, United States). Sequence alignments were performed visually using the Genetic Data Environment (2.6) program (Smith et al., 1994). The nucleotide sequences of the pt. *rbc*L gene were aligned based on the translated amino acid sequences.

### Phylogenetic analyses

**FIGURE 5:**
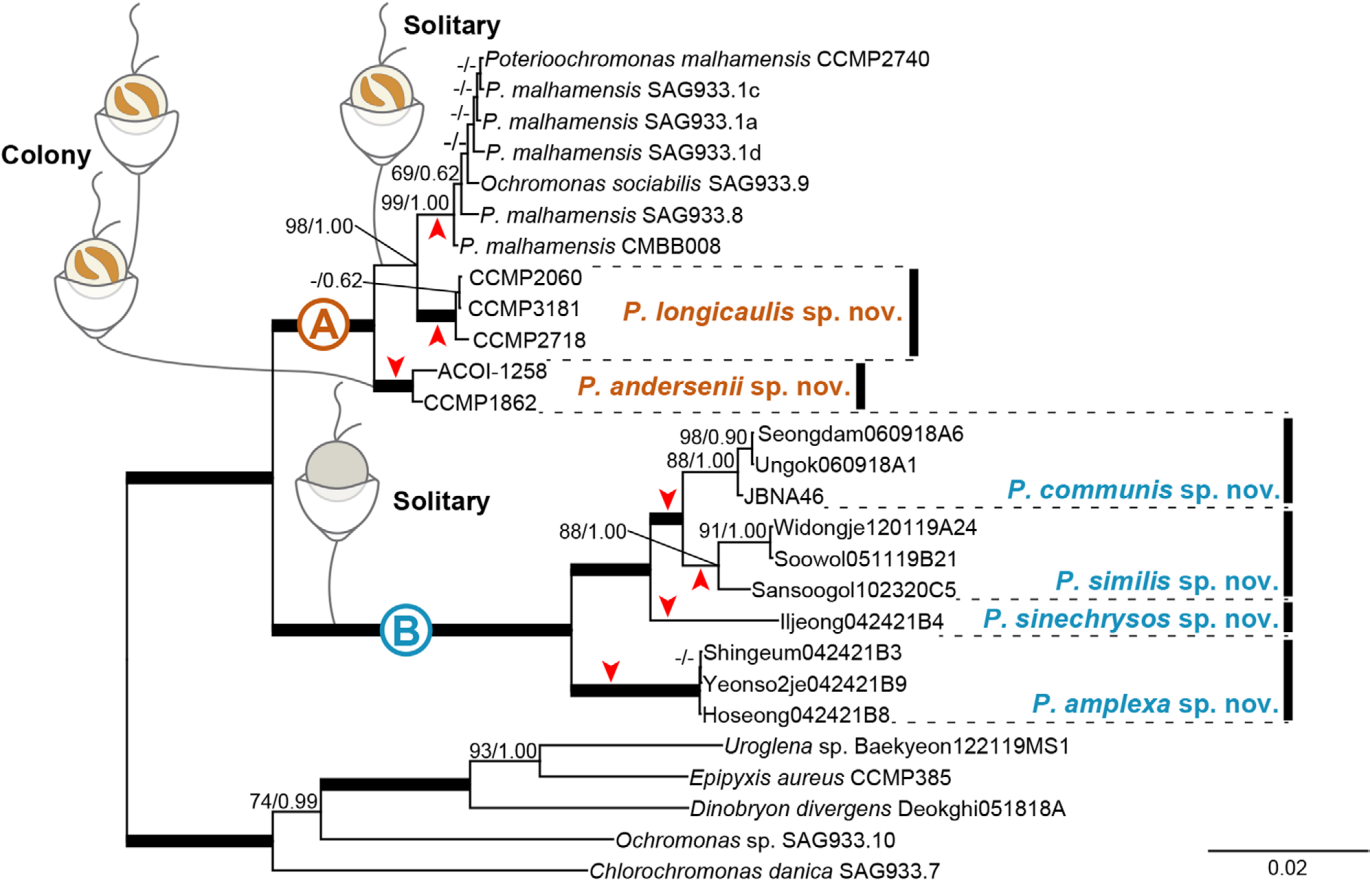
Consensus Bayesian tree of the genus *Poterioochromonas* based on the combined nuclear SSU rRNA gene, LSU rRNAgenen, ITS rRNA region and plastid LSU rRNA gene, *rbc*L gene sequence data. The maximum‐likelihood bootstrap values (MLBS values, left) and Bayesian posterior probabilities (PP, right) are shown at each node. The arrowhead below or above each node indicates each lineage; the scale bar indicates the number of substitutions/site; the thick line indicates full support (100% MLBS and 1.00 PP), and (−) denotes values <50% for MLBS or 0.50 for PP.

The phylogenetic tree of Chrysophyceae was constructed by using 1591 nucleotides of the nr SSU rRNA gene from 126 chrysophycean taxa (Figure S1). The phylogenetic analysis of *Poterioochromonas* species was performed using four genes and the ITS rRNA region with a combined dataset of 7461 nucleotides (nr SSU rRNA gene = 1641 bp, nr ITS rRNA region = 478 bp, nr LSU rRNA gene = 2369 bp, pt. LSU rRNA gene = 2274 bp, pt. *rbc*L gene = 699 bp) from 22 *Poterioochromonas* taxa (Figure 5). Only the conserved regions of the nucleotide sequences were used for phylogenetic analyses, and ambiguously aligned regions were excluded. The maximum‐likelihood (ML) analysis was performed using RAxML version 8.2.10 (Stamatakis, 2014) with the substitution model GTR + Γ. We generated 1000 independent tree inferences, using the # option of the program to identify the best tree. Maximum‐likelihood bootstrap (MLBS) values were calculated using 1000 pseudo‐replicates with the same substitution model. Bayesian analysis was performed using MrBayes version 3.7 (Ronquist et al., 2012), and the best‐fitting model for the nucleotide dataset was selected using the Bayesian information criterion in jModelTest2 (Posada & Crandall, 1988) with the selected GTR + I + Γ model. Each analysis was performed using a Metropolis‐coupled Markov chain Monte Carlo (MC^3^) approach, with 10,000,000 cycles for each chain. Trees were saved to a file every 1000 cycles, and the burn‐in point was identified graphically by tracking the likelihoods (Tracer version 1.7.1; http://tree.bio.ed.ac.uk/software/tracer/). The first 3000 trees were discarded, and the remaining 7001 trees were used to calculate the posterior probability (PP) of each clade. The trees were visualized using FigTree v.1.4.4 (http://tree.bio.ed.ac.uk/software/figtree/).

## RESULTS

### Taxonomic descriptions

#### *Poterioochromonas* Scherffel 1901, emend. M. Jeong, J.I. Kim & W. Shin

DESCRIPTION: Cell is housed in hemispherical or conical‐shaped loricae, more or less long, fine stalk, and relatively thickened foot or sometimes exhibits free‐swimming behavior. The type of lifestyle is either solitary or colonial. The vegetative cell has two unequal flagella and exhibits an amoeboid stage with feeding‐baskets. Most photosynthetic or non‐photosynthetic species use feeding‐baskets at the front of the cell to ingest various types of prey. Photosynthetic species have one to three plastids.
Type species: *Poterioochromonas stipitata* Scherffel

#### *Poterioochromonas andersenii* M. Jeong, J.I. Kim & W. Shin sp. nov.

DESCRIPTION: Circular cells are housed in hemispherical cup‐shaped loricae within an organized colony or exhibit free‐swimming behavior (Figure 1a–f). One daughter lorica attaches to the foot near the opening of the mother lorica (Figure 1a,b). The vegetative cell has two unequal flagella and one to two plastids (Figure 1d–f) and exhibits an amoeboid stage with feeding‐baskets (Figure 1f). The cell is 5.4–6.9 μm diam., 5.3–7.3 μm in length. The lorica is 4.4–8.0 μm in cup opening width, 3.3–6.7 μm in cup depth, and 23.0–115.0 μm in stalk length (*n* = 25).
10 helix of V2 region from nr SSU rRNA: ACCCU
Loop region in 10 helix of V2 region from nr SSU rRNA: UGACUUCUGGAA
10′ helix of V2 region from nr SSU rRNA: AGGGU
E23‐5 helix of V4 region from nr SSU rRNA: GCAGU
E23‐5′ helix of V4 region from nr SSU rRNA: ACUGU
HOLOTYPE: NNIBR OR410, a permanent microscope slide prepared from strain CCMP1862, deposited in the Nakdonggang National Institute of Biological Resources, Sangju, Korea (NNIBR).
ISOTYPE: NNIBR OR410, metabolically inactive specimens dried on filter paper (47 mm diam).
Reference strain: CCMP1862, deposited in the National Center for Marine Algae and Microbiota (https://ncma.bigelow.org/)
TYPE LOCALITY: A *Sphagnum* bog near Thayer Lake, Sherman Township, Newaygo County, Michigan, USA (47.275496, −88.262395), collected by Dr. R.A. Andersen, 16 Aug. 1984
ETYMOLOGY: The species is named after Dr. Robert A. Andersen, who collected and established the culture of CCMP1862.

#### *Poterioochromonas longicaulis* M. Jeong, J.I. Kim & W. Shin sp. nov.

DESCRIPTION: Circular cells are housed in hemispherical cup‐shaped loricae within an organized colony or exhibit free‐swimming behavior (Figure 1g–k). The vegetative cell has two unequal flagella and one to two plastids (Figure 1g–h). The cell is 6.6–10.6 μm in width, 6.4–11.0 μm in length. The lorica is 5.2–10.0 μm in cup opening width, 3.9–10.6 μm in cup depth, and 24.6–49.4 μm in stalk length (*n* = 25).
10 helix of V2 region from nr SSU rRNA: ACCCU
Loop region in 10 helix of V2 region from nr SSU rRNA: UGACUUCUGGAA
10′ helix of V2 region from nr SSU rRNA: AGGGU
E23‐5 helix of V4 region from nr SSU rRNA: GCAGC
E23‐5′ helix of V4 region from nr SSU rRNA: GCUGU
HOLOTYPE: NNIBR OR412, a permanent microscope slide prepared from strain CCMP2060, deposited in the Nakdonggang National Institute of Biological Resources, Sangju, Korea (NNIBR)
ISOTYPE: NNIBR OR413, metabolically inactive specimens dried on filter paper (47 mm diam.).
Reference strain: CCMP2060, deposited in the National Center for Marine Algae and Microbiota (https://ncma.bigelow.org/)
TYPE LOCALITY: Southwest shore of Man‐of‐War Cay, Smithsonian Carrie Bow Cay Field Station, Belize, Central America (16.80272, −88.08192), collected by Dr. R.A. Andersen, 06 Feb. 1987
ETYMOLOGY: The Latin specific epithet “*longicaulis*” was derived from Latin longi‐ (= long) and caulis (= stalk of the lorica).

#### *Poterioochromonas amplexa* M. Jeong, J.I. Kim & W. Shin sp. nov.

DESCRIPTION: Circular cells are housed in hemispherical cup‐shaped loricae or exhibit free‐swimming behavior (Figure 2a–f). The vegetative cell has two unequal flagella and exhibits an amoeboid stage with feeding‐baskets (Figure 2c–f), and is 3.8–10.8 μm in width, 3.8–10.0 μm in length. The lorica is 4.5–10.7 μm in cup opening width, 4.2–11.1 μm in cup depth, and 17.9–49.5 μm in stalk length (*n* = 25).

#### *Poterioochromonas communis* M. Jeong, J.I. Kim & W. Shin sp. nov.

DESCRIPTION: Circular, oblate, or widely elliptic cells are housed in hemispherical cup‐shaped loricae or exhibit free‐swimming behavior (Figure 2g–l). The vegetative cell has two unequal flagella and exhibits an amoeboid stage with feeding‐baskets (Figure 2i–l) and is 4.9–8.6 μm in width, 4.3–7.5 μm in length. The lorica is 4.4–5.6 μm in cup opening width, 3.7–5.7 μm in cup depth, and 19.7–31.1 μm in stalk length (*n* = 25).
10 helix of V2 region from nr SSU rRNA: ACUCC
Loop region in 10 helix of V2 region from nr SSU rRNA: UGACUUCUGGAA
10′ helix of V2 region from nr SSU rRNA: GGAGU
E23‐5 helix of V4 region from nr SSU rRNA: GCGGU.
E23‐5′ helix of V4 region from nr SSU rRNA: ACUUGC
HOLOTYPE: NNIBR OR414, a permanent microscope slide prepared from strain Ungok060918A1, deposited in the Nakdonggang National Institute of Biological Resources, Sangju, Korea (NNIBR)
ISOTYPE: NNIBR OR415, metabolically inactive specimens dried on filter paper (47 mm diam.)
Reference strain: A living material was deposited at the Korean Microalgae Culture Collection in the Pukyong National University, Busan, South Korea as strain KMCC‐9002
TYPE LOCALITY: Daehab‐myeon, Changnyeong‐gun, Gyeongsangnam‐do, Korea (35.629321, 128.448853), 09 Jun. 2018
ETYMOLOGY: The Latin specific epithet “*communis*” refers to the ordinary shape of the cell.

#### *Poterioochromonas similis* M. Jeong, J.I. Kim & W. Shin sp. nov.

DESCRIPTION: Circular or oblate cells are housed in hemispherical cup‐shaped loricae or exhibit free‐swimming behavior (Figure 2m–q). The vegetative cell has two unequal flagella and exhibits an amoeboid stage with feeding baskets (Figure 2o,q) and is 3.5–6.9 μm in width, 3.5–6.6 μm in length. The lorica is 2.9–8.7 μm in cup opening width, 2.3–6.2 μm in cup depth, and 23.4–33.3 μm in stalk length (*n* = 25).
10 helix of V2 region from nr SSU rRNA: ACUCC
Loop region in 10 helix of V2 region from nr SSU rRNA: UGACUUCUGGAA
10′ helix of V2 region from nr SSU rRNA: GGAGU.
E23‐5 helix of V4 region from nr SSU rRNA: GCGGU
E23‐5′ helix of V4 region from nr SSU rRNA: ACUUGU
HOLOTYPE: NNIBR OR416, a permanent microscope slide prepared from strain Widong120119A24, deposited in the Nakdonggang National Institute of Biological Resources, Sangju, Korea (NNIBR)
ISOTYPE: NNIBR OR417, metabolically inactive specimens dried on filter paper (47 mm diam.)
Reference strain: A living material was deposited at the Korean Microalgae Culture Collection (KMCC) in the Pukyong National University, Busan, South Korea as strain KMCC‐9003.
TYPE LOCALITY: Deoksan‐ri, Seongnae‐myeon, Gochang‐gun, Jeollabuk‐do, Korea (35.560588, 126.730328), 01 Dec. 2019
ETYMOLOGY: The Latin specific epithet “*similis* (= similar)” refers to the shape being extremely similar to that of other non‐photosynthetic *Poterioochromonas* species.

#### *Poterioochromonas sinechrysos* M. Jeong, J.I. Kim & W. Shin, sp. nov.

DESCRIPTION: Circular or oblate cells are housed in hemispherical cup‐shaped loricae or exhibit free‐swimming behavior (Figure 2r–v). The vegetative cell has two unequal flagella and exhibits an amoeboid stage with feeding‐baskets (Figure 2t,v), and is 4.8–8.0 μm in width, 4.5–8.9 μm in length. The lorica is 2.6–7.0 μm in cup opening width, 3.3–6.8 μm in cup depth, and 14.8–34.7 μm in stalk length (*n* = 25).
10 helix of V2 region from nr SSU rRNA: ACUCC
Loop region in 10 helix of V2 region from nr SSU rRNA: UGACUUCUGGAA
10′ helix of V2 region from nr SSU rRNA: GGAGU
E23‐5 helix of V4 region from nr SSU rRNA: GCGGU
E23‐5′ helix of V4 region from nr SSU rRNA: ACCUGU
HOLOTYPE: NNIBR OR418, a permanent microscope slide prepared from strain Iljeong042421B4, deposited in the Nakdonggang National Institute of Biological Resources, Sangju, Korea (NNIBR)
ISOTYPE: NNIBR OR419, metabolically inactive specimens dried on filter paper (47 mm diam.)
Reference strain: A living material was deposited at the Korean Microalgae Culture Collection (KMCC) in the Pukyong National University, Busan, South Korea as strain KMCC‐9004.
TYPE LOCALITY: Seokjeong‐ri, Geumsan‐myeon, Goheung‐gun, Jeollanam‐do, Korea (34.467296, 127.149872), 24 Apr. 2021
ETYMOLOGY: The specific epithet “*sinechrysos*” was derived from Latin sine‐ (= without) and chrysos (= gold; the color of the chrysophycean plastid).

### Light and fluorescence microscopy

The lorica was composed of the hemispherical‐ or conical‐shaped cup, the stalk, and the foot attaching to substratum (Figures 1a–d,g–i,l–n and 2a,b,g,h,m,n,r,s). Among seven *Poterioochromonas* taxa, six species had a solitary lifestyle, whereas *P. andersenii* exclusively formed a colony lined up as a row (Figure 1a,b). The daughter lorica of *P. andersenii* attached its foot to the cup region of the mother lorica (Figure 1a,b). The stalk region of the lorica in *P. andersenii* exhibited the longest stalk length (23 to 115 μm) compared to the other six *Poterioochromonas* species (*P. amplexa*, *P. communis*, *P. longicaulis*, *P. malhamensis*, *P. similis*, *P. sinechrysos*). One to three plastids were observed in three photosynthetic taxa of *P. andersenii*, *P. longicaulis* and *P. malhamensis* (Figure 1). The red autofluorescences from plastids were seated in the blue‐stained cup region of the lorica (Figure 1c,g,h,l,m). However, plastid and red autofluorescence in four non‐photosynthetic species, *P. amplexa*, *P. communis*, *P. similis* and *P. sinechrysos*, were not observed (Figure 2). The non‐photosynthetic *Poterioochromonas* species sometimes attached to substratum using pseudopodium without lorica (Figure 2d,j).

Feeding behavior involving the consumption of bacteria was observed in *Poterioochromonas* species, regardless of the presence or absence of plastids (Figures 1k and 2f,l,p,u). The long flagellum caused water currents for detecting a bacterium in the contact phase. After a food particle was detected, the long flagellum pressed the bacterium closely to the cell body in the processing phase. Then, the bacterium was ingested into the cell body using feeding‐baskets in the ingestion phase (Figure 3). The feeding‐basket exhibited two types of protrusions, a pronounced extension or shallow bulge (Figure 3). Finally, protrusion of the cell body involved in phagocytosis returned, and the hunted bacterium was positioned in the food vacuole of the cell in the refractory phase.

### Stomatocyst

*Poterioochromonas amplexa* formed a stomatocyst with a distinctive complex collar structure in culture conditions. The stomatocyst body was a smooth and spherical and measured 6.5–8.5 μm in length and 6.5–8.5 μm in width (*n* = 14). The cylindrical primary collar was surrounded by a secondary collar, which is composed of a cylindrical base and two or three curved projections (Figure 4). These projections converged at the apical end.

### Phylogenetic analyses

The phylogenetic tree of 126 chrysophycean taxa based on nr SSU rRNA gene sequences was divided into 10 orders (Figure S1). Seven orders were well supported as a monophyletic lineage within the chrysophytes, and Chrysosphaerales was a single‐isolate lineage including *Chrysastrella breviappendiculata*. Chromulinales formed a monophyletic lineage but was weakly supported (MLBS = 61, PP = 0.98). Paraphysomonadales was not highly supported as a monophyletic lineage (MLBS = 46, PP = 0.77). Ochromonadales was supported as a monophyletic clade with high support values (MLBS = 96, PP = 1.00) including the genus *Poterioochromonas*. The genus *Poterioochromonas* formed a monophyletic clade with strong support values (MLBS = 100, PP = 1.00) and grouped together with genera *Urostipulosphaera* and *Acrispumella* with weak support values (MLBS = 26, PP = 0.58).

The phylogenetic tree of *Poterioochromonas* taxa based on five genes divided into two major clades [A] and [B], congruent with the presence or absence of plastids (Figure 5). The A clade included three mixotrophic species that possess plastids, *P. malhamensis*, *P. longicaulis*, and *P. andersenii*. *P. malhamensis* formed sister relationships with *P. longicaulis* with high support values (MLBS = 98, PP = 1.00). Within the A clade, the colonial species *P. andersenii* was separated from two solitary species, *P. malhamensis* and *P. longicaulis*. The B clade was strongly supported (MLBS = 100, PP = 1.00) and composed of four species, *P. communis, P. similis, P. sinechrysos*, and *P. amplexa* that lack plastids. Within the B clade, *P. communis* grouped together with *P. similis* with strong support values (MLBS = 100, PP = 1.00). *Poterioochromonas sinechrysos* formed a single culture strain and grouped together with *P. communis* and *P. similis* with strong support values (MLBS = 100, PP = 1.00). *Poterioochromonas amplexa* grouped together with *P. communis, P. similis*, and *P. sinechrysos* with strong support values (MLBS = 100, PP = 1.00).

### Molecular signature

**FIGURE 6:**
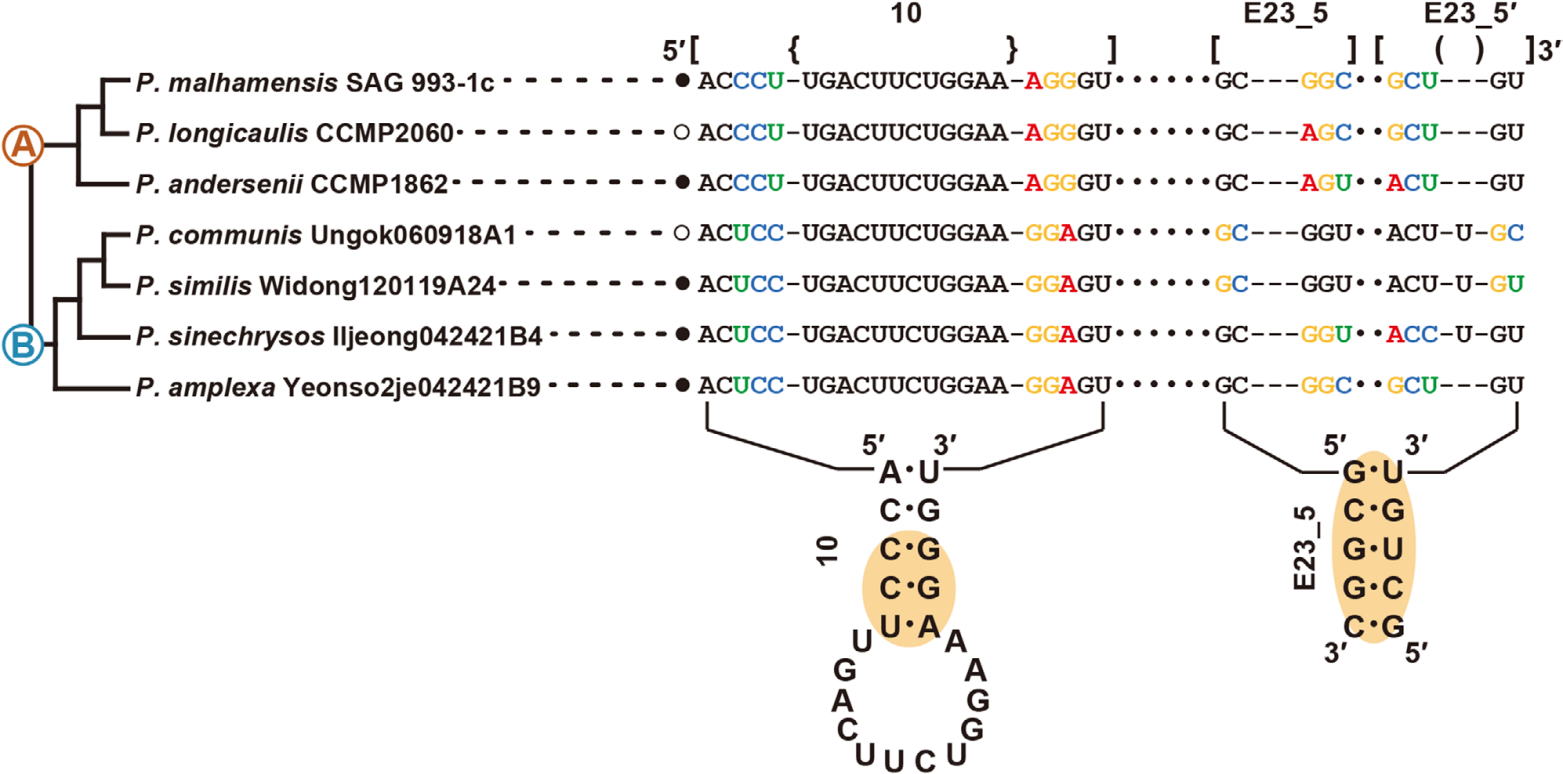
Molecular signatures of the 10 helix of the V2 region and the E23‐5 helix of the V4 region in the nuclear SSU rRNA gene differentiating the *Poterioochromonas* species. The secondary structure was constructed based on the rRNA molecules of *P. malhamensis* SAG 933‐1c. The nomenclature of nucleotides and base pairs depends on the polarity of the DNA: Increasing numbers generally indicate the 5′ to 3′ direction. [ ] indicates the beginning and end of the stem, { } indicates the beginning and end of the loop, ( ) indicates bulge of the stem.

The sequences of the 10 helix of the V2 region and E23‐5 helix of the V4 region from the nr SSU rRNA gene were selected as molecular signatures for the species delimitation of the genus *Poterioochromonas* (Figure 6). *Poterioochromonas* species each had a specific molecular signature, and the signatures of each species were compared with those of closely related species. In the stem region of helix 10, the compensatory base pair changes (CBCs) occurred between photosynthetic (clade A; 5′‐CCU:AGG‐3′) and non‐photosynthetic (clade B; 5′‐UCC:GGA‐3′) *Poterioochromonas* species. Nucleotide differences among *Poterioochromonas* species were found in E23‐5 helix of the V4 region. For example, within clade A the species *P. malhamensis* had different base pairs ‘5'‐GGC:GCU‐3'’, compared to the ‘5'‐AGC:GCU‐3'’ base pairs of the closely related species *P. longicaulis*. *Poterioochromonas andersenii* had different based pairs ‘5'‐AGU:ACU‐3'’, compared to those of *P. longicaulis* and *P. malhamensis*. Within B clade, the base pairs ‘5'‐GC:GC‐3'’ of *P. communis* were different with base pair ‘5'‐GC:GU‐3'’ of closely related species *P. similis*. The species *P. sinechrysos* had different base pairs ‘5'‐GGU:ACC‐3'’, compared to those of *P. communis*, *P. similis* (‘5'‐GGU:ACU‐3'’) and *P. amplexa* (‘5'‐GGC:GCU‐3'’).

## DISCUSSION

### Morphology of *Poterioochromonas* and closely related genera

The genus *Poterioochromonas* inhabits freshwater environments and is known as photosynthetic algae, but it is one of the taxa that is very rarely reported. Since Scherffel (1901) established this genus, only three species with plastids have been described, each characterized by a unique lorica morphology and nutritional mode. Paradoxically, despite its unique lorica shape, the extent of the biodiversity of *Poterioochromonas* remains difficult to uncover, as this is not clearly observable under a light microscope. In this study, we collected samples from various aquatic environments and established multiple non‐photosynthetic culture strains. Our morphological and molecular phylogenetic studies of both non‐photosynthetic and photosynthetic taxa revealed greater diversity within *Poterioochromonas* than we initially expected. To observe their morphological characteristics, we used fluorescence microscopy. The photosynthetic and non‐photosynthetic species differed in their nutrition modes, solitary or colonial lifestyle, presence or absence of plastid, lorica dimension, and stalk length (Table 1). Furthermore, these organisms form a feeding‐basket, as described by Scherffel, which they use to ingest prey (Figure 3). Molecular phylogeny based on a concatenated five gene sequence dataset revealed that colorless species grouped together with photosynthetic *Poterioochromonas* species as a monophyletic clade within the Ochromonadales. Consequently, based on both morphological characters and molecular phylogeny, we propose that this colorless Chrysophycean species inhabiting a lorica represents a newly discovered non‐photosynthetic *Poterioochromonas* species.

**TABLE 1:** Summary of morphological characteristics of *Poterioochromonas* species.

Taxon	Strain	Lifestyle	Plastid	Nutritional mode	Cell morphology			Lorica morphology		
					Shape	Width (μm)	Length (μm)	Cup opening width (μm)	Cup depth (μm)	Stalk length (μm)
*Poterioochromonas andersenii* ^1^	CCMP1862	Colony	+	Mixotroph	Circular	5.4–6.9	5.3–7.3	4.4–8.0	3.3–6.7	23.0–115.0
*Poterioochromonas longicaulis* ^1^	CCMP2060	Solitary	+	Mixotroph	Circular, Oblate, Widely elliptic	6.6–10.6	6.4–11.0	5.2–10.0	3.9–10.6	24.6–49.4
*Poterioochromonas malhamensis* ^1^	CCMP2740	Solitary	+	Mixotroph	Circular, Widely elliptic	5.1–7.8	4.8–8.4	4.2–9.8	5.2–7.5	16.4–42.8
*Poterioochromonas nutans* ^2^	Unknown	Solitary	+	Photoautotroph	Circular	7.0–7.6 in diameter		8.0–9.0	7.0–8.0	Up to 30.0
*Poterioochromonas stipitata* ^3,4^	Unknown	Solitary	+	Mixotroph	Circular	7.0–10.0 in diameter			Up to 20.0	
*Poterioochromonas amplexa* ^1^	Yeonso2je042421B9	Solitary	−	Heterotroph	Circular	3.8–10.8	3.8–10.0	4.5–10.7	4.2–11.1	17.9–49.5
*Poterioochromonas communis* ^1^	Ungok060918A1	Solitary	−	Heterotroph	Circular, Oblate, Widely elliptic	4.9–8.6	4.3–7.5	4.4–5.6	3.7—5.7	19.7–31.1
*Poterioochromonas similis* ^1^	Widong120119A24	Solitary	−	Heterotroph	Circular, Oblate	3.5–6.9	3.5–6.6	2.9–8.7	2.3–6.2	23.4–33.3
*Poterioochromonas sinechrysos* ^1^	Iljeong042421B4	Solitary	−	Heterotroph	Circular, Oblate	4.8–8.0	4.5–8.9	2.6–7.0	3.3–6.8	14.8–34.7

*Note*: (+) indicates presence of plastid and (−) indicates absence of plastid (^1^This study; ^2^Jane, 1944; ^3^Pascher, 1913; ^4^Scherffel, 1901).

Among Chrysophycean taxa, there are several colorless genera of solitary or colonial forms that are loricated; three solitary genera, *Arthochrysis*, *Arthropyxis*, *Stokesiella*, and four colonial genera, *Codonodendron*, *Conodobotrys*, *Dinobryon*, *Stephanocodon* (Ettl, 1968; Kent, 1880; Lemmermann, 1910; Pascher, 1930, 1942; Skuja, 1939; Stokes, 1885a, 1885b). In 1942, Pascher erected two solitary‐form genera that are morphologically very similar to *Poterioochromonas*: *Arthrochrysis* and *Arthropyxis*. Both genera share the common feature of having a lorica similar to that of *Poterioochromonas*, but the difference between these two genera is the presence or absence of plastids. The genus *Arthrochrysis* bearing plastid includes two species: *A. leptopus* and *A. pallida*. The genus *Arthropyxis* includes one colorless species, originally described by Skuja (1939) as *Stokesiella annulata*, which was transferred to *Arthropyxis* as a type. However, these two genera are distinguished from *Poterioochromonas* by having somewhat narrow lorica openings and two to three ring‐shaped transverse ridges located below the middle part of the lorica.

In 1910, Lemmermann established the genus *Stokesiella*, including five species previously described by Stokes in the genus *Bicosoeca* (Stokes, 1885a, 1885b). The morphological characteristics of this genus are very similar to the colorless *Poterioochromonas*. However, unlike that of *Poterioochromonas*, the lorica of *Stokesiella* tends to narrow slightly at the opening, and its length is approximately more than twice its width, except for *S. acuminata*.

Additionally, less than half of the cells of *Poterioochromonas* species are enclosed within a hemispherical or conical‐shaped lorica cup, while in *Stokesiella* species, two‐thirds or more, or even the entire cell, is enclosed within a vase‐ or cylindrical‐shaped lorica cup. Therefore, there is a difference between the two genera in how much of the cell is enclosed by the lorica cup. According to Pascher (1930), three species—*S. disimilis, S. epipyxis, S. lepteca—*have a stigma in front of the cell, differentiating them from *Poterioochromonas* species.

Among the loricate chrysophytes, the colonial forms include *Codonodendron*, *Conodobotrys*, *Dinobryon*, and *Stephanocodon* (Ehrenberg, 1834; Pascher, 1942). In the case of *Dinobryon*, the opening of the lorica is quite wide, and the part immediately below tends to gradually narrow, then widens again in the middle before tapering to a pointed base (vase‐shaped lorica). Additionally, *Dinobryon* does not have a stalk connected to the lorica. Another loricate genus very similar to *Dinobryon* is *Codonodendron* (Pascher, 1942). Unlike *Dinobryon*, this genus is not free‐swimming; instead, the base of the lorica, located at the very bottom of the colony, attaches to a substrate, and it is characterized by having a single flagellum. The colonial *Codonobotrys* attaches to the substrate by a foot and a main stalk. Several elliptical or cylindrical‐shaped loricae with a short stalk are attached to the upper part of this main stalk, forming an umbrella‐like shape. *Stephanocodon* has an elliptical‐shaped lorica and forms a radial arrangement without a stalk or foot. Therefore, colonial loricate chrysophytes are distinguished based on the colony arrangement, the lorica morphology, and the presence or absence of a stalk.

### Taxonomy of *Poterioochromonas* species

The previously known three species of *Poterioochromonas* have one to three plastids and exhibit a solitary lifestyle (Jane, 1944; Péterfi, 1969; Pringsheim, 1952; Scherffel, 1901). According to the original illustrations of the type species (Scherffel, 1901: figures 7, 8), *P. stipitata* has a lorica with a cup depth significantly greater than the width of the cup opening, giving it a cone‐shaped appearance. This unique lorica morphology is the most notable difference compared to the other species analyzed in this study. *Poterioochromonas nutans*, described by Jane (1944), possesses a goblet‐shaped lorica similar to other species but is distinguished by having the shortest stalk (~30 μm in length), which is sometimes comparable in length to the cup depth (8–9 μm). Therefore, these two previously known species are distinguished from the others included in this study by their unique lorica morphology and short stalk length. Additionally, both species are known to have a conical region between the base of the lorica and the part where the stalk connects to it, forming a septum that transverses the base of the lorica. However, none of the species observed in this study under fluorescence microscopy exhibited a septum, a finding consistent with Péterfi's (1969) observations.

*Poterioochromonas malhamensis* was transferred from the genus *Ochromonas* by Péterfi (1969) based on the observation that *O. malhamensis* possesses a goblet‐shaped lorica and a stalk. The authentic culture of *O. malhamensis* is known to be SAG933.1a, isolated by Dr. Chen. Additionally, the authentic culture of another new *Ochromonas* species isolated by Pringsheim, *O. sociabilis* (SAG933.9) was also included in the molecular phylogenetic analysis of this study. Our phylogenetic analysis strongly supports that *P. malhamensis* forms a monophyletic clade and suggests Pringsheim's *O. sociabilis* (SAG933.9) should be considered a synonym of *P. malhamensis*.

In this study, we have described a new colony‐forming species, *Poterioochromonas andersenii* sp. nov., which originates from the previously established strain CCMP1862. According to Andersen et al. (2017), the morphology of the strain CCMP1862 is similar to *P. nutans* in terms of plastid numbers and lorica shape, or is similar to *P. stipitata* and *P. malhamensis* in terms of cell dimension and its habit. The most notable feature of CCMP1862 observed under fluorescence microscopy was that a single daughter lorica was attached to the upper part of the mother lorica, forming a single row arrangement. Moreover, our phylogenetic analyses showed that *P. andersenii* CCMP1862 is distinctively separated from two solitary species, *P. malhamensis* and *P. longicaulis*. We also have described a second new species, *P. longicaulis* sp. nov., originating from the previously established strain CCMP2060. The lorica cup opening width of *P. longicaulis* is nearly equal to its cup depth, giving the lorica a hemispherical appearance. This characteristic differentiates *P. longicaulis* from *P. stipitata*. Additionally, *P. longicaulis* can be distinguished from *P. nutans* based on the length of the lorica stalk and the presence of food vacuoles. The lorica stalk of *P. longicaulis* is significantly longer, ranging from 24.6 to 49.4 μm, compared to that of *P. nutans*. Moreover, while *P. nutans* lacks food vacuoles, *P. longicaulis* exhibits prominent food vacuoles within the cell (see Figure 1k). Therefore, the two new species, *P. andersenii* CCMP1862 and *P. longicaulis* CCMP2060, are clearly differentiated from the previously known *Poterioochromonas* species based on molecular data, morphological characters, and colony formation.

The newly discovered heterotrophic *Poterioochromonas* species are well distinguished from previously known species based on both the absence of plastid and molecular data. Heterotrophic *Poterioochromonas* species are divided into four subclades, exhibiting genetic variations. All four lineages commonly had a circular vegetative cell shape, feeding behavior using a feeding basket, and an amoeboid stage. Additionally, they all had a lorica composed of a cup, stalk, and a foot with similar dimensions among them (Figure S2). *Poterioochromonas* species not only exhibited a wide range of morphological variation within a species but also showed morphological similarities between different species. For example, the rounded cells of *P. malhamensis* ranged from 6 to 8 μm diam. (Péterfi, 1969). However, according to Chen et al. (2020), the cells of *P. malhamensis* range from 6.0 to 12.4 μm diam. These cell dimensions overlap with those of *P. andersenii* (5.4–6.9 μm width × 6.2–7.3 μm length), *P. longicaulis* (6.8–10.6 μm width × 7.1–11.0 μm length), and *P. malhamensis* (5.0–7.8 μm width × 5.0–8.4 μm length). Furthermore, the cell dimension of *P. stipitata* (7.0–10.0 μm) described by Pascher (1913) overlaps with those of other species. In the case of the cup dimensions of *P. malhamensis*, Péterfi (1969) described it as 4.0–8.0 μm width × 3.2–5.0 μm depth, whereas Chen et al. (2020) reported it as 4.0–11.3 μm width × 3.2–10.7 μm depth. These cup dimensions also overlap with those of *P. andersenii* (4.4–8.0 μm width × 3.3–8.5 μm depth), *P. longicaulis* (5.3–13.0 μm width × 3.9–10.7 μm depth) and *P. malhamensis* (4.2–9.8 μm width × 5.2–9.4 μm depth). Moreover, the stalk length of *P. malhamensis* has significant variations, ranging from 10.0 to 129.9 μm (Chen et al., 2020). Therefore, the vegetative cell morphology of *Poterioochromonas* species does not provide a reliable taxonomic criterion for species delimitation.

### Molecular signature

The molecular signatures of two helices from the nr SSU rRNA gene data were investigated to overcome the limitations of species delineation solely based on morphological characteristics. Since identifying microalgal species based solely on morphological characteristics is very difficult, molecular signatures have been efficiently used for the delimitation of species (Škaloudova & Škaloud, 2013; Kim et al., 2013a, 2013b, 2017; Jeong et al., 2021, 2023). In particular, the E23‐5 helix of the V4 region from the nr SSU rRNA gene has been a useful genetic character for delimiting Chrysophycean species having ambiguous and simple vegetative cell morphology (Jeong et al., 2021, 2023). Furthermore, the 10 helix of the V2 region from the nr SSU rRNA gene was largely congruent with the phylogenetic relationships between photosynthetic and non‐photosynthetic species. Therefore, the delimitation of *Poterioochromonas* species can be easily evaluated using specific molecular signatures of the combination of the 10 helix of the V2 region and the E23‐5 helix of the V4 region from nr SSU rRNA gene sequences.

### Stomatocyst

The formation of the stomatocyst has been reported in many Chrysophycean genera, and its ultrastructure is known for a critical feature that offers more reliable species delimitation than vegetative cell morphology in various Chrysophycean species (Pascher, 1914; Krieger, 1930; Lund, 1942; Hilliard & Asmund, 1963; Nygaard, 1979; Duff et al., 1995; Holen, 2014; Jeong et al., 2021, 2023; Pusztai & Škaloud, 2022; Wilkinson et al., 2001). Among previously known *Poterioochromonas* species, the stomatocyst of *P. malhamensis* was described using electron microscope (Chen et al., 2020) and stomatocyst images of *P. longicaulis* CCMP2060 are available on the NCMA website. In this study, only one non‐photosynthetic species, *P. amplexa*, formed stomatocysts under culture conditions. Three species, *P. longicaulis, P. malhamensis*, and *P. amplexa*, commonly had a true complex collar structure with a cylindrical primary collar.

However, in the illustration of *Poterioochromonas malhamensis* (Ma et al., 2018), the primary collar is covered by a cap, making the pore appear laterally open. The secondary collar surrounds the primary collar with three short inward‐curving projections. The third, short collar surrounds all of these structures. In contrast, *P. longicaulis* and *P. amplexa* do not have a cap‐covered pore. Interestingly, in *P. amplexa*, the apical ends of two to three secondary collars converge to form a dome shape. The stomatocyst morphology of *P. longicaulis* CCMP2060, as recorded on the NCMA website, exhibits a short, simple cylindrical primary collar. The secondary collar surrounding the primary collar is not clearly visible on the website, but it is presumed to be present. The most distinctive feature is the tertiary collar, which has two to three broad, latitudinally positioned, lobe‐like projections. This unique collar structure differentiates *P. longicaulis* from both *P. amplexa* and *P. malhamensis*.

## CONCLUSIONS

In this study, we performed a taxonomic study of *Poterioochromonas* species based on molecular and morphological evidence. We offered detailed images of lorica morphology. All of the *Poterioochromonas* species produced the lorica, and a colonial species, *P. andersenii*, was newly described. Notably, we have also observed the diversity of non‐photosynthetic *Poterioochromonas* species. The non‐photosynthetic *Poterioochromonas* species exhibit high genetic diversity, and we describe the four new non‐photosynthetic *Poterioochromonas* species. This study demonstrated that the genus *Poterioochromonas* comprises both photosynthetic and non‐photosynthetic species, showcasing a wide range of species diversity.

## AUTHOR CONTRIBUTIONS

**Minseok Jeong:** Data curation (equal); formal analysis (equal); visualization (equal); writing – original draft (equal); writing – review and editing (equal). **Jong Im Kim:** Data curation (equal); funding acquisition (lead); writing – original draft (equal); writing – review and editing (equal). **Woongghi Shin:** Conceptualization (equal); formal analysis (equal); funding acquisition (equal); writing – original draft (equal); writing – review and editing (equal).

## FUNDING INFORMATION

National Research Foundation (NRF) of Korea, Grant Number: 2019R1I1A2A01063159, 2021R1C1C2012996, 2022R1A6A3A13067741. Korea Environment Industry & Technology Institute (KEITI), Grant Number: 2021003420004.

## Supporting information


**Figure S1.** Consensus Bayesian tree of the Chrysophyceae based on nr SSU rRNA gene sequence data. The maximum‐likelihood bootstrap values (MLBS values, left) and Bayesian posterior probabilities (PP, right) are shown at each node. The scale bar indicates the number of substitutions/site; the thick line indicates full support (100% MLBS and 1.00 PP), and (−) denotes values <50% for MLBS or 0.50 for PP.

**Figure S2.** Morphology analysis of cell and lorica dimensions of *Poterioochromonas* species.

**Table S1.** Strain information of genus *Poterioochromonas* used in this study and the GenBank accession numbers for their nuclear SSU, LSU rDNA, ITS rDNA, plastid LSU rDNA, *rbc*L gene sequences. The bold letters indicate newly obtained sequences in this study.
