# Supplementary figures and images for: Taxonomy of the genus Poterioochromonas (Chrysophyceae) based on morphological and molecular evidence
Source: J Phycol. 2025 May 26;61(3):607–22. doi: 10.1111/jpy.70028 (PMC12168102; doi:10.1111/jpy.70028)

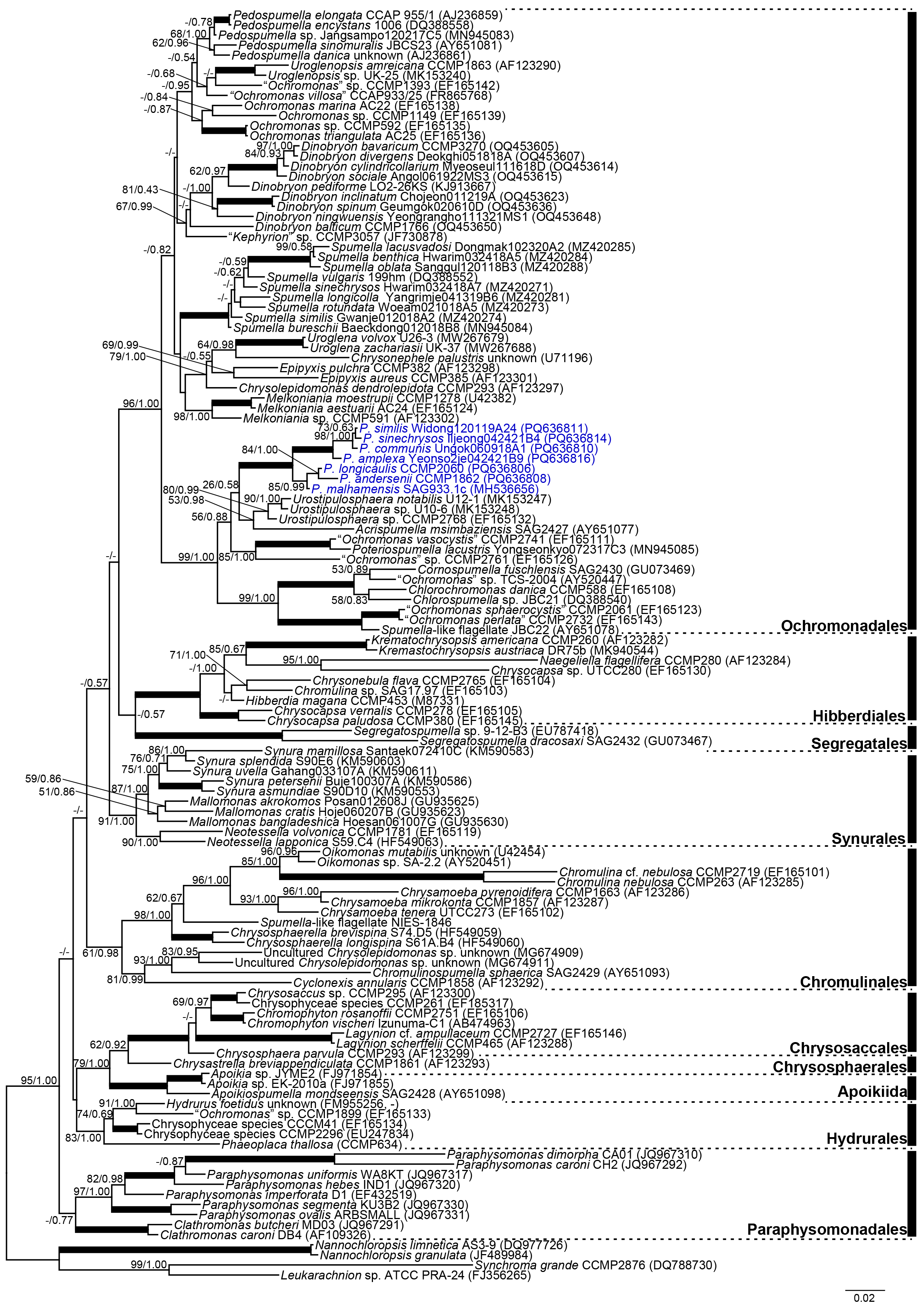

Supplement: Supplementary file 1 — Figure S1. Consensus Bayesian tree of the Chrysophyceae based on nr SSU rRNA gene sequence data. The maximum‐likelihood bootstrap values (MLBS values, left) and Bayesian posterior probabilities (PP, right) are shown at each node. The scale bar indicates the number of substitutions/site; the thick line indicates full support (100% MLBS and 1.00 PP), and (−) denotes values <50% for MLBS or 0.50 for PP. [file JPY-61-607-s003.tif]

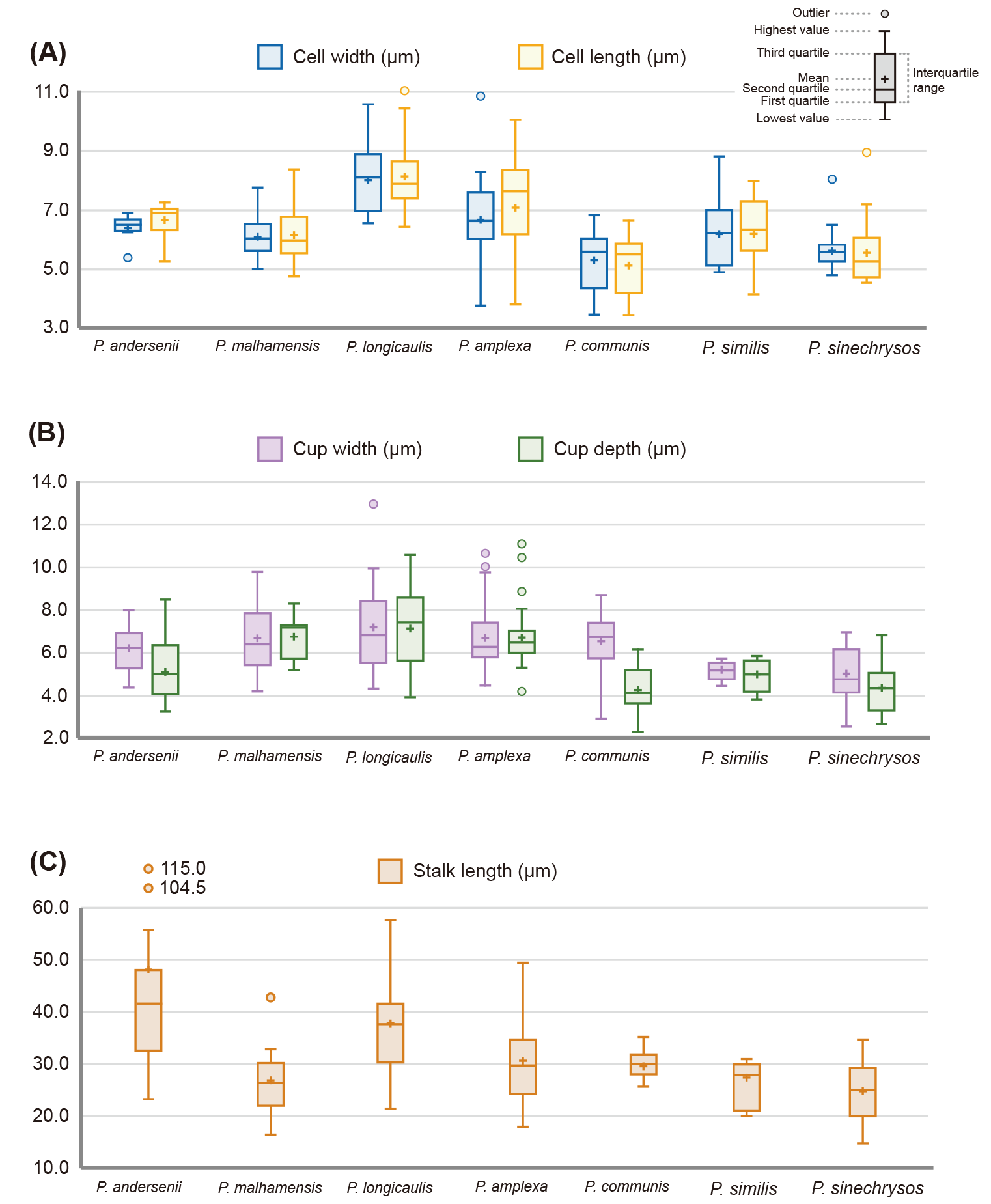

Supplement: Supplementary file 2 — Figure S2. Morphology analysis of cell and lorica dimensions of Poterioochromonas species. [file JPY-61-607-s001.tif]
