# Supplementary material for: Taxonomy of the genus Poterioochromonas (Chrysophyceae) based on morphological and molecular evidence
Source: J Phycol. 2025 May 26;61(3):607–22. doi: 10.1111/jpy.70028 (PMC12168102; doi:10.1111/jpy.70028)
Supplement: Supplementary file 3 — Table S1. Strain information of genus Poterioochromonas used in this study and the GenBank accession numbers for their nuclear SSU, LSU rDNA, ITS rDNA, plastid LSU rDNA, rbcL gene sequences. The bold letters indicate newly obtained sequences in this study. [file JPY-61-607-s002.docx]

**Supplementary Table1.** Strain information of genus *Poterioochromonas* used in this study and the GenBank accession numbers for their nuclear SSU, LSU rDNA, ITS, plastid LSU rDNA, *rbc*L gene sequences. The bold letters indicate newly obtained sequences in this study.

| Taxon | Strain | Collection site (GPS coordinates ) | Genbank accession number | | | | |  |
| --- | --- | --- | --- | --- | --- | --- | --- | --- |
|  |  |  | nr SSU | nr ITS | nr LSU | pt LSU | pt *rbc*L |  |
| ***Poterioochromonas*** | | | | | | | | |
| *P. amplexa* | Hoseong042421B8 | Seongchon-ri, Goheung-eup, Goheung-gun, Jeollanam-do, Korea (34.607396, 127.255149) | **PQ636817** | **PQ636847** | **PQ636860** | **PQ636832** | - |  |
| *P. amplexa* | Shingeum042421B3 | Shinchon-ri, Geumsan-myeon, Goheung-gun, Jeollanam-do, Korea (34.480101, 127.122486) | **PQ636815** | **PQ636845** | **PQ636858** | **PQ636830** | - |  |
| *P. amplexa* | Yeonso2je042421B9 | Eojeon-ri, Geumsan-myeon, Goheung-gun, Jeollanam-do, Korea (34.443386, 127.121251) | **PQ636816** | **PQ636846** | **PQ636859** | **PQ636831** | - |  |
| *P. andersenii* | ACOI-1258 | Unknown | DQ388542 | - | - | - | - |  |
| *P. andersenii* | CCMP1862 | A *Sphagnum* bog near the Thayer Lake Sherman, Township, Michigan, America (47.275496, -88.262395) | **PQ636808** | **PQ636838** | **PQ636851** | **PQ636823** | **PQ634385** |  |
| *P. longicaulis* | CCMP2060 | Southwest shore of Man-of-War Cay, Smithsonian Carrie Bow Cay Field Station, Belize, Central America (16.80272, -88.08192) | **PQ636806** | **PQ636836** | **PQ636849** | **PQ636821** | **PQ634383** |  |
| *P. longicaulis* | CCMP2718 | Darby River, Victoria, Australia (-38.98, 146.28) | EF165112 | - | - | - | EF165170 |  |
| *P. longicaulis* | CCMP3181 | near Port Campbell, Victoria, Australia (-38.6475, 143.0567) | **PQ636807** | **PQ636837** | **PQ636850** | **PQ636822** | **PQ634384** |  |
| *P. malhamensis* | CCMP2740 | Rhode Island, United States of America | **PQ636805** | **PQ636835** | **PQ636848** | **PQ636820** | **PQ634382** |  |
| *P. malhamensis* | CMBB008 | Unknown | MH536660 | - | - | - | MH643619 |  |
| *P. malhamensis* | SAG933.1a | Malham Tarm, Yorkshire Dales National Park, England (54.096354, -2.164221) | MH536656 | MH536656 | - | - | MH643685 |  |
| *P. malhamensis* | SAG933.1c | England | EF165114 | GU935635 | MH49141 | KM818001 | EF165169 |  |
| *P. malhamensis* | SAG933.1d | Massachusetts, Yale Univ., Osborn Lab. (41.316378, -72.923829) | MH536659 | MH536659 | - | - | MH643689 |  |
| *P. malhamensis* | SAG933.8 | Tübingen, from culture of *Paramecium bursaria* (48.524791, 9.057426) | MH542676 |  | - | - | MH643687 |  |
| *P. malhamensis* | SAG933.9 | Stadpark, Bad Driburg, Germany (51.72965, 9.022479) | MH542675 | - | - | - | MH643686 |  |
| *P. communis* | Ungok060918A1 | Daehab-myeon, Changnyeong-gun, Gyeongsangnam-do, Korea (35.629321, 128.448853) | **PQ636810** | **PQ636840** | **PQ636853** | **PQ636825** | - |  |
| *P. communis* | Seongdam060918A6 | Myeong-ri, Gyeseong-myeon, Changnyeong-gun, Gyeongsangnam-do, Korea (35.474815, 128.514069) | **PQ636809** | **PQ636839** | **PQ636852** | **PQ636824** | - |  |
| *P. similis* | Sansoogol102320C5 | Gaejeon-ri, Hoengseong-eup, Hoeongseong-gun, Gangwon-do, Korea (37.501240, 128.007482) | **PQ636813** | **PQ636843** | **PQ636856** | **PQ636828** | - |  |
| *P. similis* | Soowol051119B21 | Anseong-ri, Daejeong-eup, Seoguipo-si, Jeju-do, Korea (33.258251, 126.278457) | **PQ636812** | **PQ636842** | **PQ636855** | **PQ636827** | - |  |
| *P. similis* | Widongje120119A24 | Deoksan-ri, Seongnae-myeon, Gochang-gun, Jeollabuk-do, Korea (35.560588, 126.730328) | **PQ636811** | **PQ636841** | **PQ636854** | **PQ636826** | - |  |
| *P. sinechrysos* | Iljeong042421B4 | Seokjeong-ri, Geumsan-myeon, Goheung-gun, Jeollanam-do, Korea (34.467296, 127.149872) | **PQ636814** | **PQ636844** | **PQ636857** | **PQ636829** | - |  |
| **Outgroup** |  |  |  |  |  |  |  |  |
| *Chlorochromonas danica* | SAG 933.7 | Bog pool, Everdrup, Denmark (55.203321, 11.946394) | JQ281514 | - | GU935636 | KM590767 | GU935657 |  |
| *Dinobryon divergens* | Deokghi051818A | Deokghi-dong. Iksan-si, Jeollabuk-do, Korea  (35.978249, 127.035306) | **OQ453607** | - | **OQ453328** | **OQ453557** | **OQ466165** |  |
| *Epipyxis aureus* | CCMP385 | Sequoyah Pond, near Sequoyah Lake, Fayetteville, USA (36.039898, -94.050019) | **PQ636819** | - | **PQ636862** | **PQ636834** | **PQ634387** |  |
| *Ochromonas* sp. | SAG 933.10 | Pond, Solling, Germany (51.722778, 09.621667) | EF165109 | - | GU935637 | KM590768 | GU935658 |  |
| *Uroglena* sp. | Baekyeon122119MS1 | Daeji-ri, Donggang-myeon, Naju-si, Jeollanam-do, Korea (34.957083, 126.549056) | **PQ636818** | - | **PQ636861** | **PQ636833** | **PQ634386** |  |
